# Enhanced recovery protocols in patients undergoing pancreatic surgery: An umbrella review

**DOI:** 10.1002/nop2.923

**Published:** 2021-06-09

**Authors:** Jing Li, Frances Lin, Shuhui Yu, Andrea P. Marshall

**Affiliations:** ^1^ Nursing department Peking University First Hospital Beijing China; ^2^ School of Nursing, Midwifery, and Paramedicine University of the Sunshine Coast Maroochydore DC QLD Australia; ^3^ Sunshine Coast Health Institute Birtinya QLD Australia; ^4^ School of Nursing and Midwifery Griffith University Southport QLD Australia; ^5^ Urological Ward Peking University First Hospital Beijing China; ^6^ Nursing and Midwifery Education and Research Unit Gold Coast Health Southport QLD Australia

**Keywords:** enhanced recovery, pancreatic surgery, umbrella review

## Abstract

**Aim:**

To identify, synthesize and appraise the systematic reviews of ERAS for patients undergoing pancreatic surgery and facilitate ERAS implementation.

**Design:**

An umbrella review was used to identify systematic reviews.

**Methods:**

A systematic search following the PRISMA guidelines was used to search databases including PubMed, Embase, Cochrane Library, CINAHL, CNKI, WanFang and VJIP. AMSTAR 2 was used to appraise the quality of included reviews.

**Results:**

Ten systematic reviews were included. The quality of all included systematic reviews was rated as “critically low.” The most frequently reported ERAS elements were epidurals analgesia/PCA (9/10), goal‐directed mobilization (9/10) and early removal of drains (9/10). Only one review mentioned audit protocol compliance. None of the included reviews reported discharge standards. Ten reviews reported decreased length of stay, seven reviews reported lower hospital costs, and six reviews reported decreased total complications rate. There were no adverse effects reported.

## 1 INTRODUCTION

Enhanced recovery after surgery (ERAS), also known as “fast track,” “critical pathways” or “clinical pathways” (Coolsen et al., 2013), is a multimodal, multidisciplinary approach to perioperative care which was introduced to improve patient outcomes and reduce healthcare costs (Ljungqvist et al., 2017). ERAS protocols have developed rapidly in the last decade and focussed on bundle elements including, but not limited to, preoperative information and teaching, decreased of stress, pain relief, early mobilization and early oral diet (Bond‐Smith et al., 2016; Ljungqvist et al., 2017). ERAS guidelines have been developed for elective rectal/pelvic surgery (Nygren et al., 2012), radical cystectomy for bladder cancer (Cerantola et al., 2013), oesophagectomy (Findlay et al., 2014) and gastrectomy (Mortensen et al., 2014), and all suggest that ERAS protocols can decrease the length of hospital stay and reduce costs in these surgery procedures (Cerantola et al., 2013; Findlay et al., 2014; Mortensen et al., 2014; Nygren et al., 2012). Enhanced recovery after pancreatic surgery was developed cautiously because of the complexity of pancreatic surgery. Kennedy et al., (2007) initially reported the enhanced recovery protocol after pancreatic surgery in 2007, which reported improved patient outcomes. Coolsen et al., (2015) also reported that ERAS protocols were feasible and did not compromise patient outcomes in older adults. The first guideline for perioperative care for pancreaticoduodenectomy published in 2012 contained 27 recommendations (Lassen et al., 2012). Subsequently, further reviews on enhanced recovery after pancreatic surgery have shown that it is effective in decreasing hospital length of stay (Pecorelli et al., 2016), was less expensive and although no difference in readmission and postoperative morbidity was observed (Barton, 2016; Elhassan et al., 2019; Feng et al., 2017; Kagedan et al., 2015; Pecorelli et al., 2016; Perinel & Adham, 2016; Xie et al., 2016; Ypsilantis & Praseedom, 2009).

However, existing reviews (Coolsen et al., 2013; Kagedan et al., 2015; Xie et al., 2016; Ypsilantis & Praseedom, 2009) demonstrated variability in the ERAS elements. Outcome indicators are inconsistently reported, which lead to uncertainty for clinical decision makers, and made clinical application challenging. The aim of this umbrella review was to identify, synthesize and appraise the systematic reviews on enhanced recovery after pancreatic surgery and provide critical analysis of ERAS interventions and outcome measures, and to facilitate ERAS implementation.

## 2 REVIEW METHODS

### 2.1 Design

This is an umbrella review. The screening process followed the Preferred Reporting Items for Systematic Reviews and Meta‐Analyses (PRISMA) guideline (Moher et al., 2009). The articles were identified from target databases according to the search strategy and were exported into Endnote X9 (Clarivate Analytics, Philadelphia PA). Duplicates were removed. The title and abstract of all articles were initially screened according to inclusion and exclusion criteria; full text screening against the inclusion and exclusion criteria followed.

### 2.2 Search methods

A search strategy (Table 1) was developed with the support of professors of surgery and research librarians. PubMed, Excerpta Medica Database (EMBASE), Cochrane Library, Cumulative Index of Nursing and Allied Health Literature (CINAHL), China National Knowledge Infrastructure (CNKI), Wan Fang and VIP Journal integration platform (VJIP) were searched from inception to 1 October 2019. Reference lists of included reviews were searched to locate additional reviews. Websites of the International Association of Pancreatology.

**TABLE 1:** Search strategy (in Pubmed)

#1 pancreaticoduodenectomy[MeSH Terms] #2 pancreatectomy[MeSH Terms] #3 pancreatic*[Title/Abstract] #4 #1 OR #2 OR #3 #5 “recovery of function”[MeSH Terms] #6 “enhanced recovery after surgery”[Title/Abstract] #7 ERAS[Title/Abstract] #8 “enhanced recovery”[Title/Abstract] #9 “fast track surgery”[Title/Abstract] #10 fast‐track[Title/Abstract] #11 FT[Title/Abstract] #12 #5 OR #6 OR #7 OR #8 OR #9 OR #10 OR #11 #13 #4 AND #12 #14 Search (#4 AND #12) Filters: Review; Scientific Integrity Review; Systematic Reviews; Publication date to 2019/10/01

(http://www.internationalpancreatology.org/), American Pancreatic Association (https://www.american‐pancreatic‐association.org/) and ERAS Society (http://erassociety.org/) were also searched to identify additional systematic reviews. Papers were included if they: (1) included patients undergoing any elective pancreatic surgical procedure: pancreaticoduodenectomy (PD), distal pancreatectomy (DP), pylorus‐preserving pancreaticoduodenectomy (PPPD), segmental pancreatectomy (SP), duodenum‐preserving pancreatic head resection (DPPHR), total pancreatectomy (TP); (2) involved elements of ERAS; (3) included outcomes of ERAS; (4) used a systematic review methodology; and (5) were published in English or Chinese. Papers were excluded if they were (1) abstract of conference paper; (2) unavailable as full text; or (3) a duplicate publication.

### 2.3 Quality appraisal

The AMSTAR 2 tool (Shea et al., 2017), which contains 16 discrete evaluation questions and was designed for critical appraisal of systematic reviews, was used to guide quality assessment. Seven of the AMSTAR 2 items are described as critical domains (Item 2, 4, 7, 9, 11, 13, 15); the remaining nine items are considered non‐critical. Quality assessment is described as “high” (one or fewer non‐critical weakness), “moderate” (more than one non‐critical weakness), “low” (one critical flaw with or without non‐critical weaknesses) and “critically low” (more than one critical flaw with or without non‐critical weakness). Two authors independently undertook a quality assessment of included articles; a third author assisted with consensus moderation where required.

### 2.4 Data abstraction and synthesis

Data extraction was independently conducted by two authors. An overview of study characteristics is provided in Table 2, ERAS elements of studies included in each review are summarized in Table 3, and patient outcomes are summarized Table 4.

**TABLE 2:** Main characteristics of included reviews

Author, year, country	Methods of Study	Search Date	Language limitation	Type of surgery	Included articles	Study design of included articles	Patients in ERAS (*N*)/Control Group (*N*)	AMSTAR 2 results^*^
Ypsilantis and Praseedom (2009), UK	Systematic review	Not reported	English	Elective surgery for pancreatic malignancy	3	3 CCT	626/296	Critically low
Coolsen et al. (2013), Netherlands	Meta‐analysis	Jan 1966 to Dec 2012	English Dutch German	Elective pancreatic resection	8	7 CCT 1 Prospective case series	1090/468	Critically low
Kagedan et al., 2015), Canada	Systematic review	2000 to 2013	Not reported	Pancreatic surgery	10	9 CCT 1 Prospective case series	1129/513	Critically low
Lei et al., (2015), China	Meta‐analysis	Jan 1991 to May 2014	English Chinese	PD	14	8 CCT 6 RCT	1366/1199	Critically low
Xiong et al., (2016), China	Meta‐analysis	Jan 2000 to Jun 2015	English	PD	14	14 CCT	1409/1310	Critically low
Xie et al., (2016), China	Meta‐analysis	To Oct 2015	No restriction	Pancreatic surgery	16	15 CCT 1 Prospective case series	2016/1030	Critically low
Zhang et al., (2018), China	Meta‐analysis	To Sep 2016	English Chinese	PD	16	12 CCT 4 RCT	1401/1427	Critically low
Ji et al., (2018), China	Meta‐analysis	Jan 1995 to Aug 2017	English	Pancreatic surgery	20	20 CCT	1886/1808	Critically low
Chen et al., (2019), China	Meta‐analysis	To Mar 2018	Not reported	PD	10	5 CCT 5 RCT	773/906	Critically low
Cao et al., (2019), China	Meta‐analysis	To May 2018	Not reported	PD, PPPD, PJ, proximal/ distal pancreatic resection	19	19 CCT	1766/1621	Critically low

Note

RCT, Randomized Controlled trial; CCT, Clinical Controlled trial; PD, pancreaticoduodenectomy; PJ, pancreaticojejunostomy; PPPD, pylorus‐preserving pancreaticoduodenectomy.

* Details of the critical assessment are available in Table S1.

**TABLE 3:** Summary of ERAS elements in included reviews

Included review	Preoperative elements				Intraoperative elements								Postoperative elements								Total number of elements listed in the SR
	Preoperative counselling	No oral bowel preparation	Preoperative clear fluids	Antithrombotic prophylaxis	No preanaesthetic medication	Preoperative antibiotics	Prevention of postoperative nausea and vomiting	Avoiding hypothermia	Perioperative glycaemic control	Epidurals analgesia/PCA	Early removal of nasogastric tube	Maintaining fluid balance	Goal‐directed mobilization	Early oral intake	Early removal of Foley catheter	Early removal of drains	Prokinetic agents	Octreotides	Discharge planning	Audit	
Ypsilantis and Praseedom (2009)	√	—	—	√	—	√	—	—	—	√	√	—	√	√	√	√	√	√	√	—	12
Coolsen et al. (2013)	√	√	√	√	√	√	√	√	√	√	√	√	√	√	√	√	√	√	—	√	19
Kagedan et al. (2015)	—	—	—	—	—	√	—	—	—	√	√	—	—	—	√	√	√	√	√	—	8
Lei et al., (2015)	—	—	—	—	—	√	—	—	—	√	—	—	√	√	—	√	√	—	—	—	6
Xiong et al., (2016)	—	—	—	—	—	√	—	—	—	√	√	—	√	√	√	√	√	√	√	—	10
Xie et al., (2016)	—	—	—	—	—	√	—	—	—	√	√	—	√	√	√	√	√	√	√	—	10
Zhang et al., (2018)	√	—	√	—	—	√	—	√	—	—	√	√	√	√	√	√	—	—	—	—	10
Ji et al., (2018)	—	√	√	—	—	—	—	—	—	√	—	√	√	√	—	√	—	—	—	—	7
Chen et al., (2019)	—	—	√	—	—	—	√	√	√	√	√	—	√	√	√	√	—	√	√	—	12
Cao et al., (2019)	√	√	√	√	√	√	√	√	√	√	√	√	√	—	—	—	—	√	—	—	14
Total reported	4	3	5	3	2	8	3	4	3	9	8	4	9	8	7	9	6	7	5	1	

Note

√: reported in the review;—: not reported in the review

**TABLE 4:** Summary of patient outcomes indicators in included reviews

Included review	LOS	Cost	Readmission rate	Reoperation rate	Mortality	Total complication morbidity	Delayed gastric emptying	Pancreatic fistula	Time of first flatulence	Abdominal infection	Biliary fistula	Incisional wound infection	Pulmonary infection
Ypsilantis and Praseedom (2009)	↓	↓	ND	—	ND	↑	—	—	—	—	—	—	—
Coolsen et al., (2013)	↓	↓	ND	—	ND	↓	ND	—	—	—	—	—	—
Kagedan et al. (2015)	↓	↓	ND	—	ND	ND	—	—	—	—	—	—	—
Lei et al., (2015)	↓	↓	ND	ND	↓	↓	—	—	—	—	—	—	—
Xiong et al., (2016)	↓	↓	ND	ND	ND	↓	↓	ND	—	—	—	—	—
Xie et al., (2016)	↓	↓	ND	—	ND	↓	↓	↓	—	—	—	—	—
Zhang et al., (2018)	↓	—	ND	ND	ND	—	↓	ND	—	—	↓	—	—
Ji et al., (2018)	↓	—	—	—	ND	↓	↓	ND	—	↓	—	—	—
Chen et al. (2019)	↓	—	—	—	—	↓	—	—	↓	—	—	—	—
Cao et al. (2019)	↓	↓	ND	ND	ND	—	ND	↓	↓	ND	—	↓	↓
Total reported	10	7	8	4	9	8	6	5	2	2	1	1	1

Note

↓: lower in the ERAS group; ↑: higher in the ERAS group, ND: No differences; —: not reported in the review.

## 3 RESULTS

### 3.1 The characteristics of included reviews

**FIGURE 1:**
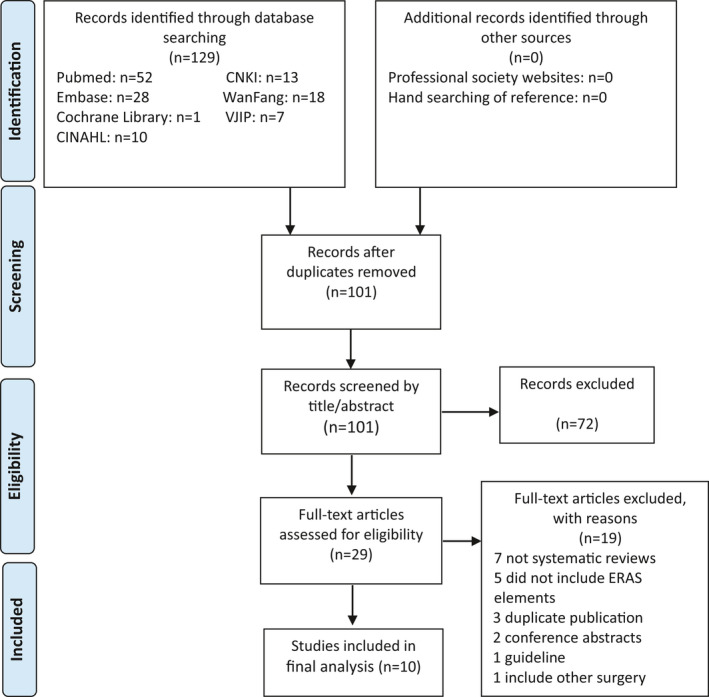
PRISMA flow diagram

A total of 10 systematic reviews met the inclusion criteria (Figure 1). Of the 10 systematic reviews included, seven reviews were from China (three were published in Chinese, and four were published in English) (Cao et al., 2019; Chen et al., 2019; Ji et al., 2018; Lei et al., 2015; Xie et al., 2016; Xiong et al., 2016; Zhang et al., 2018); there was one review each from UK, Netherlands and Canada, respectively. Two systematic reviews did not include a meta‐analysis (Kagedan et al., 2015 Ypsilantis & Praseedom, 2009). Only three reviews included randomized controlled trials (Chen et al., 2019; Lei et al., 2015; Zhang et al., 2018) (Table 2).

### 3.2 The quality of included reviews

Quality appraisal using AMSTAR2 (Shea et al., 2017) identified that all included reviews had more than one item identified as a critical weakness; thus, the quality of all the reviews was deemed as “critically low” (Table S1). Critical domains of AMSTAR2 items 2 (Did the report of the review contain an explicit statement that the review methods were established prior to the conduct of the review and did the report justify any significant deviations from the protocol?) and item 13 (Did the review authors account for Risk of Bias in primary studies when interpreting/discussing the results of the review?) were not present for any included review.

### 3.3 The ERAS elements and outcome indicators reported

The ERAS elements mentioned in the included reviews varied widely (Table 3). The most frequently reported ERAS elements were epidurals analgesia/ Patient Controlled Analgesia (PCA) (*n* = 9) (Cao et al., 2019; Chen et al., 2019; Coolsen et al., 2013; Ji et al., 2018; Kagedan et al., 2015; Lei et al., 2015; Xie et al., 2016; Xiong et al., 2016; Ypsilantis and Praseedom, 2009), goal‐directed mobilization (*n* = 9) (Cao et al., 2019; Chen et al., 2019; Coolsen et al., 2013; Ji et al., 2018; Lei et al., 2015; Xie et al., 2016; Xiong et al., 2016; Ypsilantis & Praseedom, 2009; Zhang et al., 2018) and early removal of drains (*n* = 9) (Chen et al., 2019; Coolsen et al., 2013; Ji et al., 2018; Kagedan et al., 2015; Lei et al., 2015; Xie et al., 2016; Xiong et al., 2016; Ypsilantis & Praseedom, 2009; Zhang et al., 2018). Only one review (Coolsen et al., 2013) mentioned audit protocol compliance. None of the included reviews reported discharge standards. The most commonly reported outcome was length of stay (*n* = 10) (Cao et al., 2019; Chen et al., 2019; Coolsen et al., 2013; Ji et al., 2018; Kagedan et al., 2015; Lei et al., 2015; Xie et al., 2016; Xiong et al., 2016; Ypsilantis & Praseedom, 2009; Zhang et al., 2018), mortality (*n* = 9) (Cao et al., 2019; Coolsen et al., 2013; Ji et al., 2018; Kagedan et al., 2015; Lei et al., 2015; Xie et al., 2016; Xiong et al., 2016; Ypsilantis & Praseedom, 2009; Zhang et al., 2018), total complications morbidity (*n* = 8) (Cao et al., 2019; Coolsen et al., 2013; Ji et al., 2018; Kagedan et al., 2015; Lei et al., 2015; Xie et al., 2016; Xiong et al., 2016; Ypsilantis & Praseedom, 2009) and readmission rate (*n* = 8) (Cao et al., 2019; Coolsen et al., 2013; Kagedan et al., 2015; Lei et al., 2015; Xie et al., 2016; Xiong et al., 2016; Ypsilantis & Praseedom, 2009; Zhang et al., 2018). Only two reviews (Cao et al., 2019; Chen et al., 2019) reported that ERAS resulted in a decrease in the time of first flatulence, and no review reported time to first oral intake or mobilization as well as the degree of recovery of patients at the time of discharge.

### 3.4 The effectiveness and safety of ERAS after pancreatic surgery

The effectiveness of ERAS was reported. Ten reviews reported decreased length of stay (Cao et al., 2019; Chen et al., 2019; Coolsen et al., 2013; Ji et al., 2018; Kagedan et al., 2015; Lei et al., 2015; Xie et al., 2016; Xiong et al., 2016; Ypsilantis & Praseedom, 2009; Zhang et al., 2018), seven reviews reported lower hospital costs (Cao et al., 2019; Coolsen et al., 2013; Kagedan et al., 2015; Lei et al., 2015; Xie et al., 2016; Xiong et al., 2016; Ypsilantis & Praseedom, 2009) and six reviews reported decreased total complications rate (Chen et al., 2019; Coolsen et al., 2013 Ji et al., 2018; Lei et al., 2015; Xie et al., 2016; Xiong et al., 2016). Safety of ERAS was reported as no adverse effect incidents of ERAS (Kagedan et al., 2015), no difference in mortality, (Cao et al., 2019; Coolsen et al., 2013; Ji et al., 2018; Kagedan et al., 2015; Xie et al., 2016; Xiong et al., 2016; Ypsilantis & Praseedom, 2009; Zhang et al., 2018), readmission rate (Cao et al., 2019; Coolsen et al., 2013; Kagedan et al., 2015; Lei et al., 2015; Xie et al., 2016; Xiong et al., 2016; Ypsilantis & Praseedom, 2009; Zhang et al., 2018), reoperation rate (Cao et al., 2019; Lei et al., 2015; Xiong et al., 2016; Zhang et al., 2018) and rate of pancreatic fistula (Ji et al., 2018; Xiong et al., 2016; Zhang et al., 2018). The impact of ERAS on patient outcomes is summarized in Table 4.

## 4 DISCUSSION

### 4.1 Overview

ERAS is a patient‐centred, evidence‐based approach to patient care, which includes multiple interventions designed to facilitate optimal postoperative recovery (Ljungqvist et al., 2017). ERAS in pancreatic surgery has been reported in the literature for over a decade (Kennedy et al., 2007), with the first ERAS guideline for pancreatic surgery published in 2012 (Lassen et al., 2012), and the most recent update published in 2020 (Melloul et al., 2020). Compared with other subspecialties in general surgery, ERAS uptake in pancreatic surgery has been comparatively cautious (Lei et al., 2015). This may be attributed to the fact that pancreatic surgery is a complex, highly difficult procedure, and often associated with more postoperative complications (Lei et al., 2015).

In this umbrella review, we found that 9/10 reviews published after the original ERAS guideline for pancreatic surgery (Lassen et al., 2012) were made available, this shows that the release of the guidelines has promoted the application of ERAS in patients after pancreatic surgery. Only three reviews included randomized controlled trials, and this indicates that research in this field is still lacking high‐quality evidence. Also, there may be bias on patient grouping, which made the results biased, decreasing the confidence of the conclusions of individual reviews.

### 4.2 The quality of included reviews

In this umbrella review, we found that the overall quality of the 10 included reviews was “critically low” according to AMSTAR 2 criteria (Shea et al., 2017). For item 2, no review published their protocol in advance, which may introduce publication bias, and unsatisfactory indicators may be ignored and reported, such as adverse events and unexpected withdrawal from the trial. Having an “a priori” review protocol available prior to undertaking the review is recommended (Shamseer et al., 2016) and can increase the transparency of the review process. Item 13 is another critical domain of AMSTAR 2, where no authors accounted for risk of bias in individual studies when discussing the results of the review. Risk of bias in individual studies could lead to biases on evidence synthesis in the reviews. If the conclusions of the review were applied to all patients after pancreatic surgery without analysis, it may endanger the safety of patients. Thus, these reviews do not provide the accurate and comprehensive synthesis of the available evidence (Shea et al., 2017). Clinicians should be cautious in adopting the conclusions of these systematic reviews.

### 4.3 Effectiveness and safety of ERAS after pancreatic surgery

Effectiveness and safety of enhanced recovery after pancreatic surgery were the main focus of all included reviews. All the included systematic reviews showed that ERAS after pancreatic surgery contributed to a decreased hospital stay, reducing hospital stay 0.36–4.45 days (Cao et al., 2019; Chen et al., 2019; Coolsen et al., 2013; Ji et al., 2018; Kagedan et al., 2015; Lei et al., 2015; Xie et al., 2016; Xiong et al., 2016; Ypsilantis & Praseedom, 2009; Zhang et al., 2018), lower hospital costs (Cao et al., 2019; Coolsen et al., 2013; Kagedan et al., 2015; Lei et al., 2015; Xie et al., 2016; Xiong et al., 2016; Ypsilantis & Praseedom, 2009) and decreased complication rate (Chen et al., 2019; Coolsen et al., 2013; Ji et al., 2018; Lei et al., 2015; Xie et al., 2016; Xiong et al., 2016). The effectiveness is largely due to the reduction in complications, a decreased hospital length of stay (Joliat et al., 2015), and improved quality of care through process standardization and decreased practice variation, all of which contribute to lower hospital costs (Kagedan et al., 2017). A less frequently reported effectiveness indicator was the impact of ERAS on the patient recovery process. For example, in only two reviews (Cao et al., 2019; Chen et al., 2019) was a decrease in the time of first flatulence reported, and time to first oral intake or mobilization was not reported in any review. It is suggested that more attention should be paid to observation of and reporting outcomes reflecting functional status (e.g. physical activity, activities of daily living) and overall health (e.g. quality of life) of patient recovery when ERAS is used (Feldman et al., 2015).

Safety of enhanced recovery after pancreatic surgery was also reported in the included reviews where there was no change in reported adverse events and or mortality (Cao et al., 2019; Chen et al., 2019; Coolsen et al., 2013; Ji et al., 2018; Kagedan et al., 2015; Xie et al., 2016; Xiong et al., 2016; Ypsilantis & Praseedom, 2009; Zhang et al., 2018), rate of readmission (Cao et al., 2019; Coolsen et al., 2013; Kagedan et al., 2015; Lei et al., 2015; Xie et al., 2016; Xiong et al., 2016; Ypsilantis & Praseedom, 2009; Zhang et al., 2018), reoperation (Cao et al., 2019; Lei et al., 2015; Xiong et al., 2016; Zhang et al., 2018) and pancreatic fistula (Ji et al., 2018; Xiong et al., 2016; Zhang et al., 2018). Unfortunately, abdominal infection (Cao et al., 2019; Ji et al., 2018) and biliary fistula (Zhang et al., 2018) were less frequently measured. Abdominal infection and biliary fistula are critical complications for ERAS of pancreatic resection and important to measure after pancreatic surgery, they will help clinicians make the judgement of patient recovery (Lassen et al., 2012; Melloul et al., 2020). In addition, none of the included reviews reported the degree of recovery of patients at the time of discharge or the standards of discharge, suggesting some outcome indicators such as complications were not monitored in hospitals. Therefore, it is suggested that long‐term follow‐up should be used to observe the complications in order to ensure the safety of ERAS after pancreatectomy.

### 4.4 Selection of ERAS elements

Some patient‐related outcomes reported in these reviews were not always included, such as delayed gastric emptying (Cao et al., 2019; Coolsen et al., 2013; Ji et al., 2018; Xie et al., 2016; Xiong et al., 2016; Zhang et al., 2018) and pancreatic fistula (Cao et al., 2019; Ji et al., 2018; Xie et al., 2016; Xiong et al., 2016; Zhang et al., 2018). This may relate to the lack of consistency in which ERAS elements were implemented in the protocols evaluated in the original articles included in the 10 systematic reviews. The ERAS components most commonly included were the use of epidurals analgesia/PCA (Cao et al., 2019; Chen et al., 2019; Coolsen et al., 2013; Ji et al., 2018; Kagedan et al., 2015; Lei et al., 2015; Xie et al., 2016; Xiong et al., 2016; Ypsilantis & Praseedom, 2009), goal‐directed mobilization (Cao et al., 2019; Chen et al., 2019; Coolsen et al., 2013; Ji et al., 2018; Lei et al., 2015; Xie et al., 2016; Xiong et al., 2016; Ypsilantis & Praseedom, 2009; Zhang et al., 2018) and early removal of drains (Chen et al., 2019; Coolsen et al., 2013; Ji et al., 2018; Kagedan et al., 2015; Lei et al., 2015; Xie et al., 2016; Xiong et al., 2016; Ypsilantis & Praseedom, 2009; Zhang et al., 2018). Most of the components were related to the postoperative phase, which was essential to promote optimal postoperative recovery. Among them, sufficient multimodal postoperative analgesia is strongly recommended as a core component for rapid recovery (Mendes et al., 2018; Partelli et al., 2016; Pecorelli et al., 2016), which has been found to reduce stress response to surgery and improving compliance to early goal‐directed mobilization. It can also bring in the recovery of gastrointestinal function and early removal of the nasogastric drain (Williamsson et al., 2015), thus speed up the postoperative recovery process. In addition, some items of ERAS were already supported by clear high‐level evidence and have a higher level of recommendation in the latest guideline, such as avoiding hypothermia, use of wound catheter, antimicrobial and thromboprophylaxis, preoperative nutritional interventions for patients with severe weight loss (Melloul et al., 2020), so priority should be given to these ERAS measures.

A protocol compliance audit was only included in one review as an ERAS intervention. (Coolsen et al., 2013) despite auditing and feedback of the ERAS protocol being identified as effective strategies in implementation studies (Bisch et al., 2018; Nelson et al., 2016), to help decrease ERAS practice variation. The latest guidelines published in 2020 also recommended that compliance should be documented as part of future trials (Melloul et al., 2020). Protocol compliance was found to be low in the postoperative phase (Maessen et al., 2007), and auditing adherence to the protocol is recommended as a standard item in ERAS implementation (Pecorelli et al., 2017). Brown and Xhaja (2018) proposed that ERAS coordinators could be well positioned to audit the ERAS process and promote protocol adherence through weekly chart audits, particularly in the early stages of implementation. These suggestions are important given it has been shown that maintaining high compliance, sustainability and improving patient outcomes is possible when ERAS guidelines are implemented (Arrick et al., 2019; Pisarska et al., 2018).

### 4.5 Limitations

There are some limitations in this umbrella review. First, we did not conduct a meta‐analysis in this review, due to heterogeneity on patients’ outcomes and unclear definition of outcomes in included systematic reviews. Secondly, because of the inconsistencies in the components implemented/reported in the included systematic reviews makes it difficult to clearly recommend which individual interventions are likely to be most beneficial. Last, because the quality of all the included reviews was low, it was difficult to draw clear conclusions to evaluate the benefit of the bundle of ERAS intervention.

## 5 CONCLUSION

In summary, the quality of existing systematic reviews on ERAS after pancreatic surgery suggests the further high‐quality research in this area is required. In addition, the quality of systematic reviews can be improved to make available higher‐quality evidence syntheses. The feasibility and effectiveness of individual ERAS elements also require further investigation so that those with the highest‐quality evidence is prioritized for implementation. Future research should incorporate economic evaluation, process evaluation and intervention compliance.

## CONFLICT OF INTEREST

The authors had no conflict of interest to declare.

## AUTHOR CONTRIBUTION

All authors contributed to the plan and methodological study discussion of the review. Jing Li and Shuhui Yu: Database search, data extraction and quality assessment. Jing Li: Writing. Andrea P. Marshall, Frances Lin and Shuhui Yu: Revision and approval of the final version.

## Supporting information

Table S1

## Data Availability

The data that support the findings of this study are available from the corresponding author upon reasonable request.

## References

[nop2923-bib-0001] Arrick, L. , Mayson, K. , Hong, T. , & Warnock, G. (2019). Enhanced recovery after surgery in colorectal surgery: Impact of protocol adherence on patient outcomes. Journal of Clinical Anesthesia, 55, 7–12. 10.1016/j.jclinane.2018.12.034 30583114

[nop2923-bib-0002] Barton, J. G. (2016). Enhanced recovery pathways in pancreatic surgery. The Surgical Clinics of North America, 96(6), 1301–1312. 10.1016/j.suc.2016.07.003 27865279

[nop2923-bib-0003] Bisch, S. P. , Wells, T. , Gramlich, L. , Faris, P. , Wang, X. , Tran, D. T. , Thanh, N. X. , Glaze, S. , Chu, P. , Ghatage, P. , Nation, J. , Capstick, V. , Steed, H. , Sabourin, J. , & Nelson, G. (2018). Enhanced Recovery After Surgery (ERAS) in gynecologic oncology: System‐wide implementation and audit leads to improved value and patient outcomes. Gynecologic Oncology, 151(1), 117–123. 10.1016/j.ygyno.2018.08.007 30100053

[nop2923-bib-0004] Shamseer, L. , Moher, D. , Clarke, M. , Ghersi, D. , Liberati, A. , Petticrew, M. , Shekelle, P. , & Stewart, L. A. (2016). Preferred reporting items for systematic review and meta‐analysis protocols (PRISMA‐P) 2015: Elaboration and explanation. BMJ (Clinical research ed.), 354, i4086. 10.1136/bmj.i4086 25555855

[nop2923-bib-0005] Bond‐Smith, G. , Belgaumkar, A. P. , Davidson, B. R. , & Gurusamy, K. S. (2016). Enhanced recovery protocols for major upper gastrointestinal, liver and pancreatic surgery. Cochrane Database of Systematic Reviews, 2, CD011382. 10.1002/14651858.CD011382.pub2 PMC8765738 26829903

[nop2923-bib-0006] Brown, D. , & Xhaja, A. (2018). Nursing perspectives on enhanced recovery after surgery. The Surgical Clinics of North America, 98(6), 1211–1221. 10.1016/j.suc.2018.07.008 30390853

[nop2923-bib-0007] Cao, Y. , Gu, H. , Huang, Z. , Wu, Y. , Zhang, Q. , Luo, J. , Zhang, C. , & Fu, Y. (2019). Impact of enhanced recovery after surgery on postoperative recovery for pancreaticoduodenectomy: pooled analysis of observational study. Frontiers in Oncology, 9, 687. 10.3389/fonc.2019.00687 31417868 PMC6683725

[nop2923-bib-0008] Cerantola, Y. , Valerio, M. , Persson, B. , Jichlinski, P. , Ljungqvist, O. , Hubner, M. , Kassouf, W. , Muller, S. , Baldini, G. , Carli, F. , Naesheimh, T. , Ytrebo, L. , Revhaug, A. , Lassen, K. , Knutsen, T. , Aarsether, E. , Wiklund, P. , & Patel, H. R. (2013). Guidelines for perioperative care after radical cystectomy for bladder cancer: Enhanced Recovery After Surgery (ERAS(®)) society recommendations. Clinical Nutrition (Edinburgh, Scotland), 32(6), 879–887. 10.1016/j.clnu.2013.09.014 24189391

[nop2923-bib-0009] Chen, J. , Liu, L. , Su, S. , Fu, W. , Lei, S. , & Zheng, S. (2019). Meta analysis of the effect of accelerated rehabilitation nursing on the rebatilitation of patients undergoing pancreaticoduodenectomy. Journal of Nursing Advancement, 34, 685–689.

[nop2923-bib-0010] Coolsen, M. M. , Bakens, M. , van Dam, R. M. , Olde Damink, S. W. , & Dejong, C. H. (2015). Implementing an enhanced recovery program after pancreaticoduodenectomy in elderly patients: Is it feasible? World Journal of Surgery, 39(1), 251–258. 10.1007/s00268-014-2782-x 25212064

[nop2923-bib-0011] Coolsen, M. M. , van Dam, R. M. , van der Wilt, A. A. , Slim, K. , Lassen, K. , & Dejong, C. H. (2013). Systematic review and meta‐analysis of enhanced recovery after pancreatic surgery with particular emphasis on pancreaticoduodenectomies. World Journal of Surgery, 37(8), 1909–1918. 10.1007/s00268-013-2044-3 23568250

[nop2923-bib-0012] Elhassan, A. , Elhassan, I. , Elhassan, A. , Sekar, K. D. , Rubin, R. E. , Urman, R. D. , Cornett, E. M. , & Kaye, A. D. (2019). Essential elements for enhanced recovery after intra‐abdominal surgery. Current Pain and Headache Reports, 23(5), 35. 10.1007/s11916-019-0772-2 31041558

[nop2923-bib-0013] Feldman, L. S. , Lee, L. , & Fiore, J. Jr (2015). What outcomes are important in the assessment of enhanced recovery after surgery (ERAS) pathways? Canadian Journal of Anaesthesia, 62(2), 120–130. 10.1007/s12630-014-0263-1 25391733

[nop2923-bib-0014] Feng, M. , Zhang, T. , & Zhao, Y. (2017). Present situation and prospect of enhanced recovery after surgery in pancreatic surgery. Zhe Jiang Da Xue Xue Bao Yi Xue Ban, 46, 666–674. 10.3785/j.issn.1008-9292.2017.12.15 PMC10396984 29658672

[nop2923-bib-0015] Findlay, J. M. , Gillies, R. S. , Millo, J. , Sgromo, B. , Marshall, R. E. , & Maynard, N. D. (2014). Enhanced recovery for esophagectomy: A systematic review and evidence‐based guidelines. Annals of Surgery, 259(3), 413–431. 10.1097/SLA.0000000000000349 24253135

[nop2923-bib-0016] Ji, H. , Zhu, W. , Wei, Q. , Wang, X. , Wang, H. , & Chen, Q. (2018). Impact of enhanced recovery after surgery programs on pancreatic surgery: A meta‐analysis. World Journal of Gastroenterology, 24(15), 1666–1678. 10.3748/wjg.v24.i15.1666 29686474 PMC5910550

[nop2923-bib-0017] Joliat, G. R. , Labgaa, I. , Petermann, D. , Hübner, M. , Griesser, A. C. , Demartines, N. , & Schäfer, M. (2015). Cost‐benefit analysis of an enhanced recovery protocol for pancreaticoduodenectomy. The British Journal of Surgery, 102(13), 1676–1683. 10.1002/bjs.9957 26492489

[nop2923-bib-0018] Kagedan, D. J. , Ahmed, M. , Devitt, K. S. , & Wei, A. C. (2015). Enhanced recovery after pancreatic surgery: a systematic review of the evidence. HPB: The official journal of the International Hepato Pancreato Biliary Association, 17(1), 11–16. 10.1111/hpb.12265 24750457 PMC4266435

[nop2923-bib-0019] Kagedan, D. J. , Devitt, K. S. , Tremblay St‐Germain, A. , Ramjaun, A. , Cleary, S. P. , & Wei, A. C. (2017). The economics of recovery after pancreatic surgery: Detailed cost minimization analysis of an enhanced recovery program. HPB: The Official Journal of the International Hepato Pancreato Biliary Association, 19(11), 1026–1033. 10.1016/j.hpb.2017.07.013 28865739

[nop2923-bib-0020] Kennedy, E. P. , Rosato, E. L. , Sauter, P. K. , Rosenberg, L. M. , Doria, C. , Marino, I. R. , Chojnacki, K. A. , Berger, A. C. , & Yeo, C. J. (2007). Initiation of a critical pathway for pancreaticoduodenectomy at an academic institution–the first step in multidisciplinary team building. Journal of the American College of Surgeons, 204(5), 917–924. 10.1016/j.jamcollsurg.2007.01.057 17481510

[nop2923-bib-0021] Lassen, K. , Coolsen, M. M. , Slim, K. , Carli, F. , de Aguilar‐Nascimento, J. E. , Schäfer, M. , Parks, R. W. , Fearon, K. C. , Lobo, D. N. , Demartines, N. , Braga, M. , Ljungqvist, O. , Dejong, C. H. , ERAS® Society, European Society for Clinical Nutrition and Metabolism, & International Association for Surgical Metabolism and Nutrition (2012). Guidelines for perioperative care for pancreaticoduodenectomy: Enhanced Recovery After Surgery (ERAS®) Society recommendations. Clinical Nutrition (Edinburgh, Scotland), 31(6), 817–830. 10.1016/j.clnu.2012.08.011 23079762

[nop2923-bib-0022] Lei, Q. , Wang, X. , Tan, S. , Wan, X. , Zheng, H. , & Li, N. (2015). Application of enhanced recovery after surgery program in perioperative management of pancreaticoduodenectomy: A systematic review. Chinese Journal of Gastrointestinal Surgery, 18(2), 143–149. 10.3760/cma.j.issn.1671-0274.2015.02.012 25656123

[nop2923-bib-0023] Ljungqvist, O. , Scott, M. , & Fearon, K. C. (2017). Enhanced recovery after surgery: A review. JAMA Surgery, 152(3), 292–298. 10.1001/jamasurg.2016.4952 28097305

[nop2923-bib-0024] Maessen, J. , Dejong, C. H. , Hausel, J. , Nygren, J. , Lassen, K. , Andersen, J. , Kessels, A. G. , Revhaug, A. , Kehlet, H. , Ljungqvist, O. , Fearon, K. C. , & von Meyenfeldt, M. F. (2007). A protocol is not enough to implement an enhanced recovery programme for colorectal resection. The British Journal of Surgery, 94(2), 224–231. 10.1002/bjs.5468 17205493

[nop2923-bib-0025] Melloul, E. , Lassen, K. , Roulin, D. , Grass, F. , Perinel, J. , Adham, M. , Wellge, E. B. , Kunzler, F. , Besselink, M. G. , Asbun, H. , Scott, M. J. , Dejong, C. , Vrochides, D. , Aloia, T. , Izbicki, J. R. , & Demartines, N. (2020). Guidelines for perioperative care for pancreatoduodenectomy: Enhanced recovery after surgery (ERAS) Recommendations 2019. World Journal of Surgery, 44(7), 2056–2084. 10.1007/s00268-020-05462-w 32161987

[nop2923-bib-0026] Mendes, D. , Ferrito, C. , & Gonçalves, M. (2018). Nursing Interventions in the enhanced recovery after surgery®: Scoping review. Revista Brasileira De Enfermagem, 71(suppl 6), 2824–2832. 10.1590/0034-7167-2018-0436 30540062

[nop2923-bib-0027] Moher, D. , Liberati, A. , Tetzlaff, J. , Altman, D. G. , & PRISMA Group (2009). Preferred reporting items for systematic reviews and meta‐analyses: The PRISMA statement. PLoS Med, 6(7), e1000097. 10.1371/journal.pmed.1000097 19621072 PMC2707599

[nop2923-bib-0028] Mortensen, K. , Nilsson, M. , Slim, K. , Schäfer, M. , Mariette, C. , Braga, M. , Carli, F. , Demartines, N. , Griffin, S. M. , Lassen, K. , & Enhanced Recovery After Surgery (ERAS®) Group (2014). Consensus guidelines for enhanced recovery after gastrectomy: Enhanced Recovery After Surgery (ERAS®) Society recommendations. The British Journal of Surgery, 101(10), 1209–1229. 10.1002/bjs.9582 25047143

[nop2923-bib-0029] Nelson, G. , Kiyang, L. N. , Crumley, E. T. , Chuck, A. , Nguyen, T. , Faris, P. , Wasylak, T. , Basualdo‐Hammond, C. , McKay, S. , Ljungqvist, O. , & Gramlich, L. M. (2016). Implementation of Enhanced Recovery After Surgery (ERAS) Across a Provincial Healthcare System: The ERAS Alberta Colorectal Surgery Experience. World Journal of Surgery, 40(5), 1092–1103. 10.1007/s00268-016-3472-7 26928854

[nop2923-bib-0030] Nygren, J. , Thacker, J. , Carli, F. , Fearon, K. C. , Norderval, S. , Lobo, D. N. , Ljungqvist, O. , Soop, M. , & Ramirez, J. & Enhanced Recovery After Surgery Society (2012). Guidelines for perioperative care in elective rectal/pelvic surgery: Enhanced Recovery After Surgery (ERAS®) Society recommendations. Clinical Nutrition (Edinburgh, Scotland), 31(6), 801–816. 10.1016/j.clnu.2012.08.012 23062720

[nop2923-bib-0031] Partelli, S. , Crippa, S. , Castagnani, R. , Ruffo, G. , Marmorale, C. , Franconi, A. M. , De Angelis, C. , & Falconi, M. (2016). Evaluation of an enhanced recovery protocol after pancreaticoduodenectomy in elderly patients. HPB, 18(2), 153–158. 10.1016/j.hpb.2015.09.009 26902134 PMC4814589

[nop2923-bib-0032] Pecorelli, N. , Capretti, G. , Balzano, G. , Castoldi, R. , Maspero, M. , Beretta, L. , & Braga, M. (2017). Enhanced recovery pathway in patients undergoing distal pancreatectomy: A case‐matched study. HPB, 19(3), 270–278. 10.1016/j.hpb.2016.10.014 27914764

[nop2923-bib-0033] Pecorelli, N. , Nobile, S. , Partelli, S. , Cardinali, L. , Crippa, S. , Balzano, G. , Beretta, L. , & Falconi, M. (2016). Enhanced recovery pathways in pancreatic surgery: State of the art. World Journal of Gastroenterology, 22(28), 6456–6468. 10.3748/wjg.v22.i28.6456 27605881 PMC4968126

[nop2923-bib-0034] Perinel, J. , & Adham, M. (2016). ERAS and pancreatic surgery: A review. Updates in Surgery, 68(3), 253–255. 10.1007/s13304-016-0406-8 27807815

[nop2923-bib-0035] Pisarska, M. , Gajewska, N. , Małczak, P. , Wysocki, M. , Major, P. , Milian‐Ciesielska, K. , Budzyński, A. , & Pędziwiatr, M. (2018). Is it possible to maintain high compliance with the enhanced recovery after surgery (ERAS) Protocol?‐A Cohort Study of 400 Consecutive Colorectal Cancer Patients. Journal of Clinical Medicine, 7(11), 412. 10.3390/jcm7110412 PMC6262379 30400342

[nop2923-bib-0036] Shea, B. J. , Reeves, B. C. , Wells, G. , Thuku, M. , Hamel, C. , Moran, J. , Moher, D. , Tugwell, P. , Welch, V. , Kristjansson, E. , & Henry, D. A. (2017). AMSTAR 2: A critical appraisal tool for systematic reviews that include randomised or non‐randomised studies of healthcare interventions, or both. BMJ (Clinical Research ed.), 358, j4008. 10.1136/bmj.j4008 PMC5833365 28935701

[nop2923-bib-0037] Williamsson, C. , Karlsson, N. , Sturesson, C. , Lindell, G. , Andersson, R. , & Tingstedt, B. (2015). Impact of a fast‐track surgery programme for pancreaticoduodenectomy. The British Journal of Surgery, 102(9), 1133–1141. 10.1002/bjs.9856 26042725

[nop2923-bib-0038] Xie, Z. , Chen, J. , & Fu, D. (2016). Enhanced recovery after pancreatic surgery: A systematic review. International Journal of Clinical and Experimental Medicine, 9(9), 17690–17702.

[nop2923-bib-0039] Xiong, J. , Szatmary, P. , Huang, W. , de la Iglesia‐Garcia, D. , Nunes, Q. M. , Xia, Q. , Hu, W. , Sutton, R. , Liu, X. , & Raraty, M. G. (2016). Enhanced recovery after surgery program in patients undergoing pancreaticoduodenectomy: A PRISMA‐Compliant Systematic Review and Meta‐Analysis. Medicine, 95(18), e3497. 10.1097/MD.0000000000003497 27149448 PMC4863765

[nop2923-bib-0040] Ypsilantis, E. , & Praseedom, R. K. (2009). Current status of fast‐track recovery pathways in pancreatic surgery. Journal of the Pancreas, 10(6), 646–650. 19890186

[nop2923-bib-0041] Zhang, T. , Yu, L. , Fan, S. , Pan, F. , Zhang, D. , He, Q. , & Lang, R. (2018). Systematic review of enhanced recovery after surgery in perioperative management of pancreaticoduodenectomy: A meta‐analysis of randomized controlled trials and non‐randomized controlled trials. Chinese Journal of Clinical Nutrition, 26, 1–8. 10.3760/cma.j.issn.1674-635X.2018.01.001

