# Supplementary material for: Enhanced recovery protocols in patients undergoing pancreatic surgery: An umbrella review
Source: Nurs Open. 2021 Jun 9;9(2):932–41. doi: 10.1002/nop2.923 (PMC8859084; doi:10.1002/nop2.923)
Supplement: Supplementary file 1 — Table S1 [file NOP2-9-932-s001.docx]

**Supplement 1: Results of quality assessment using AMSTAR 2**

| **AMSTAR 2 Item** | **Reviews** | | | | | | | | | |
| --- | --- | --- | --- | --- | --- | --- | --- | --- | --- | --- |
|  | Ypsilantis et al. ([2009](#_ENREF_49)) | Coolsen et al. ([2013](#_ENREF_11)) | Kagedan et al. ([2015](#_ENREF_18)) | Lei et al. ([2015](#_ENREF_22)) | Xiong et al. ([2016](#_ENREF_47)) | Xie et al. ([2016](#_ENREF_46)) | Zhang et al. ([2018](#_ENREF_50)) | Ji et al. ([2018](#_ENREF_16)) | Chen et al. ([2019](#_ENREF_8)) | Cao et al. ([2019](#_ENREF_6)) |
| 1. Did the research questions and inclusion criteria for the review include the components of PICO? | Yes | Yes | No | Yes | Yes | Yes | No | Yes | Yes | Yes |
| *2. Did the report of the review contain an explicit statement that the review methods were established prior to the conduct of the review and did the report justify any significant deviations from the protocol? | No | No | No | No | No | No | No | No | No | No |
| 3. Did the review authors explain their selection of the study designs for inclusion in the review? | No | No | No | Yes | No | No | No | Yes | Yes | Yes |
| *4. Did the review authors use a comprehensive literature search strategy? | Partial Yes | Partial Yes | Partial Yes | Partial Yes | Partial Yes | Partial Yes | Partial Yes | Partial Yes | Partial Yes | Partial Yes |
| 5. Did the review authors perform study selection in duplicate? | No | Yes | No | No | Yes | No | Yes | No | Yes | No |
| 6. Did the review authors perform data extraction in duplicate? | No | Yes | Yes | Yes | Yes | Yes | Yes | Yes | Yes | Yes |
| *7. Did the review authors provide a list of excluded studies and justify the exclusions? | No | Yes | No | No | No | No | No | No | No | No |
| 8. Did the review authors describe the included studies in adequate detail? | Partial Yes | Partial Yes | Partial Yes | Partial Yes | Partial Yes | Partial Yes | Partial Yes | Partial Yes | Partial Yes | Partial Yes |
| *9. Did the review authors use a satisfactory technique for assessing the risk of bias in individual studies that were included in the review? | No | Partial Yes | Partial Yes | Partial Yes | Partial Yes | No | Partial Yes | Partial Yes | No | Partial Yes |
| 10. Did the review authors report on the sources of funding for the studies included in the review? | No | No | No | No | No | No | No | No | No | No |
| *11. If meta-analysis was performed did the review authors use appropriate methods for statistical combination of results? | N/A | Yes | N/A | Yes | Yes | Yes | No | Yes | No | Yes |
| 12. If meta-analysis was performed, did the review authors assess the potential impact of ROB in individual studies on the results of the meta-analysis or other evidence synthesis? | N/A | No | N/A | No | No | No | No | Yes | No | No |
| *13. Did the review authors account for risk of bias in individual studies when interpreting in discussing the results of the review? | No | No | No | No | No | No | No | No | No | No |
| 14. Did the review authors provide a satisfactory explanation for, and discussion of, any heterogeneity observed in the results of the review? | No | Yes | No | No | No | No | No | Yes | Yes | No |
| *15. If they performed quantitative synthesis did the review authors carry out an adequate investigation of publication bias (small study bias) and discuss its likely impact on the results of the review? | N/A | No | N/A | No | No | Yes | Yes | Yes | No | No |
| 16. Did the review authors report any potential sources of conflict of interest, including any funding they received for conducting the review? | Yes | Yes | Yes | No | Yes | Yes | No | No | No | Yes |
| No. of “no” in critical domains | 4 | 3 | 3 | 4 | 4 | 4 | 4 | 3 | 6 | 4 |
| Rating overall confidence | Critically low | Critically low | Critically low | Critically low | Critically low | Critically low | Critically low | Critically low | Critically low | Critically low |

***： critical domains of AMSTAR 2; N/A - not applicable**
